# Focusing on implementation: the power of executing many small advances well

**DOI:** 10.9745/GHSP-D-13-00100

**Published:** 2013-08-14

## Abstract

Success often comes through many small, incremental, well-executed improvements.

The highly regarded business classic *Good to Great*^1^ found that one ingredient of highly successful businesses is pursuing and refining their core business year after year through incremental improvement and execution. So, too, in global public health. Yes, we have “game changers” in global health, to a degree, such as chlorhexidine for neonatal cord care, insecticide-treated bed nets, oral rehydration solution, contraceptive implants, pneumococcal vaccine, and even task shifting. But real success with these and dozens of other more modest interventions rests with persistent execution, often involving many painstaking steps.

This issue of GHSP provides some examples:

- **MCH programming.** Hodgins argues that maternal and neonatal health approaches need to shift from “grand strategies” to focus more on implementation content and quality, designed and carried out by informed, empowered managers responding to local situations.
- **Improving contraceptive method mix.** Family planning provides a good example of programming that has tended to succeed through steady execution over many years. Ross et al. describe how expanding the contraceptive method mix has contributed to that success and argues for the value of continuing to do so.
- **Provider behavior.** Providers and their behavior are key program ingredients. Kim et al. describe their methodology for improving antiretroviral service delivery in Zambia using the Standards-Based Management and Recognition approach. Their rather intensive intervention produced clear but fairly modest improvements. Provider behavior remains a realm needing more attention.
- **Family planning counseling.** Kamhawi et al. document a robust approach to reaching family planning clients, involving extensive outreach as well as provider training and job aids to improve counseling. They, too, found rather modest gains in family planning use.
- **mHealth.** Labrique et al. describe the vast programmatic landscape for potential mHealth applications. Paudel et al., as one example, document clear but modest and incremental gains from using tablet computers for the recent Nepal Demographic and Health Survey. So far, the experience with mHealth strongly suggests that advances will come not so much through any single major, transformational breakthrough, but rather through many adapted applications to address local problems, in a manner following the “diffusion of innovation” model.

At GHSP we are committed to illuminating and applauding the crucial and often hard work of program implementation, both large and small, and we will continue to spotlight it. – *Global Health: Science and Practice*
